# Progress in screening strategies for neonatal developmental dysplasia of the hip

**DOI:** 10.3389/fsurg.2022.995949

**Published:** 2022-10-26

**Authors:** Jiuhui Han, Yu Li

**Affiliations:** ^1^ Department of Pediatric Orthopaedics, The Third Hospital of Hebei Medical University, Shijiazhuang, China; ^2^ Department of Orthopaedics, Wuhan Children's Hospital, Tongji Medical College, Huazhong University of Science and Technology, Wuhan, China

**Keywords:** developmental dysplasia of the hip, hip screening, clinical examination, ultrasound, systematic screening

## Abstract

Developmental dysplasia of the hip (DDH) is the most common congenital disease of the musculoskeletal system in newborns and encompasses a disease spectrum ranging from a stable hip with a mildly dysplastic acetabulum to complete hip dislocation. Systematic screening for infant DDH has been performed for several decades all over the world and has contributed greatly to the early detection, diagnosis and treatment of DDH. However, some cases of delayed diagnosis still occur among the screened population, or conversely, overdiagnosis or overtreatment occasionally occurs. Furthermore, screening strategies for DDH are still controversial. The aim of our study was to analyze the current literature on DDH screening, paying particular attention to DDH screening strategies and their effectiveness. We searched the DDH screening literature from 1958 to 2021 in MEDLINE and other databases using PubMed. In this study, we reviewed the history of DDH screening and the progress of screening strategies and discussed the controversies regarding clinical and ultrasound screening methods with particular emphasis on the current opinions. Given the existing scientific evidence and changes in newborn DDH screening practices, universal ultrasound screening seems to be the best option for preventing late-detected cases and can be recommended as a favorable prevention strategy.

## Introduction

Developmental dysplasia of the hip (DDH), formerly known as congenital dislocation of the hip (CDH), is the most common congenital disease of the musculoskeletal system in newborns. DDH encompasses a disease spectrum ranging from a stable hip with a mildly dysplastic acetabulum to complete hip dislocation (1). The incidence is between 0.4% and 1% and varies by region, ethnicity, etc., and the incidence of complete hip dislocation is 1‰ (2, 3). If DDH is not given appropriate attention or timely treatment, it can be aggravated or worsen, which further negatively affects normal development and healthy growth in children (4, 5). Therefore, screening newborn hips is of great significance for the early detection, diagnosis and treatment of DDH as well as for prognosis prediction.

Planned screening for neonatal DDH was first performed in Sweden in the 1950s and has been performed for more than 70 years (6, 7). There are discrepancies in screening methods and scales in different countries. Even in diverse regions of the same country, the screening programs vary widely as a consequence of the diverse economic level (8). In the initial stage of DDH screening development, screenings were mainly based on physical examinations. With the emergence of ultrasound technology for neonatal hip assessments in the early 1980s, ultrasound gradually became the dominant DDH screening method. Ultrasound screening for DDH can either be selective or universal depending on whether specific groups of neonates or all neonates are assessed. Current screening strategies all have their own advantages and disadvantages and supporters, and some cases of delayed diagnosis even occur among the screened population, or conversely, overdiagnosis or overtreatment occasionally occurs. Therefore, we have reviewed the existing scientific evidence and changes in infant DDH screening practices and summarized the evolution of DDH screening strategies. We have also compared the effectiveness and socioeconomic aspects of various screening programs and defined efficient programs and accurate screening techniques in the hope of helping healthcare professionals choose the appropriate strategy for early diagnosis.

## History of DDH screening

As early as the late nineteenth century till early twentieth century some similar or identical tests based on infant hip instability, i.e., dislocated and repositionable, or repositioned and dislocatable hips, were developed by some dedicated physicians, such as Roser, Calot, Le Damany, Ortolani, Barlow, and so on for early diagnosis of DDH (9, 10). However, due to the absence of formal screening in most countries, DDH is usually diagnosed after a child starts standing and walking. Systematic neonatal DDH screening was carried out in some countries and regions in the 1960s, which greatly improved the early diagnosis of DDH. In the early 1960s, Von Rosen (6) from Sweden reported the early diagnosis of DDH through the clinical examination of hip instability by Ortolani sign in about 24,000 newborns and Barlow (11) from Britain did this by testing Barlow sign in about 9,289 newborns, and they claimed very satisfactory effects to eliminate late cases. Their achievements aroused worldwide attention and enthusiasm for the early diagnosis and early treatment of DDH. In 1969, the Ministry of Health of the United Kingdom implemented clinical screening for DDH in newborn babies nationwide, which led to good results. Many other European countries and North American countries subsequently published articles describing their specific screening methods and achievements (12–14). In the 1960s, newborns were screened for DDH through physical examination alone. An examiner used the Ortolani test to determine whether the hip was dislocated and the Barlow test to check the stability of the hip. Some scholars held the opinion that limited abduction was an important indicator of DDH and could be used as a supplement to the Barlow and Ortolani tests and that limited abduction could become clinically significant after one month of age.

In the 1960s, the backgrounds and screening strategies of screeners in various countries and regions were diverse. MacKenzie (15) published an article in 1972 summarizing the experience of DDH screening for all babies born in the northeastern region of Scotland between 1960 and 1969. First, all doctors and midwives were shown the physical hip examination methods. Infants born in the hospital were examined by pediatric registrars, and those born in peripheral hospitals or at home were examined by family doctors and midwives. All infants with abnormal findings were referred to the clinic for treatment in the third week after birth. At that time, some other countries and regions, such as Northern Ireland, Finland, Sweden, and Norway, adopted the same strategy, and the Swedish National Health and Welfare Board recommended routine DDH screening for all newborns. Hansson (16) published an article in 1,983 introducing the Swedish screening strategy used from 1973 to 1978 in obstetrics departments. It was recommended that the hip joints of all newborn infants be routinely examined twice in the ward by a pediatrician. Hansson stressed that it is important to perform the first clinical examination during the first 24h of life because hip instability may regress spontaneously during the first few days of life, and there is no guarantee that a newborn child whose hip instability recovers spontaneously will not later develop DDH. Formal screening of newborns for congenital hip abnormalities was started in Vancouver in 1964. Lehmann et al. (13) evaluated the effects of initial screenings in Vancouver in 1981 and pointed out that systematic screening can effectively reduce the rate of late diagnosis and showed that the greatest success occurred when screening was carried out by one experienced orthopedic surgeon and specially trained nurses in the community.

Since the 1970 s, the effectiveness of physical screening for all newborn hips has remained controversial due to the different false positive rates and false negative rates reported in the literature (15, 17, 18). Jones et al. (17) analyzed the screening results from the Norwich area of England during the five-year period from 1968 to 1972. Although the screenings were performed by experienced orthopedic surgeons, the results suggest that their efforts were effective in only 50% of the babies examined, and it is more likely that a large number of abnormal hips are undiagnosable at birth with the usual clinical instability tests. According to a study by Engesaeter et al. (19), 92% of patients who underwent hip replacement due to hip degeneration from potential dysplasia had undergone clinical screening after birth that showed no hip instability. In 1984, Robertson (20) analyzed articles of the past 30 years about DDH screening and pointed out that all the screening efforts had failed and that the need for surgeries due to DDH had not changed. Many clinical studies have shown that hip dislocation can be missed when the hip dislocation is irreducible, hip instability disappears soon after birth, or the instability is too mild to be detected. Furthermore, the incidence of hip dislocation was uncertain at that time and varied considerably from 0.041% to 16.8% by different screening agencies (21). Of course, in addition to the limitations of the physical screening method itself, the incidence is also affected by genetic and racial factors, the diagnostic criteria used, the experience and training of the examiners, and the age of the child at the time of examination. These negative results regarding the physical screening strategy prompted a variety of research projects, which produced new insights into screening programs.

## Ultrasound technology for hip examinations

### Introduction of ultrasound for hip examinations by graf

Before the 1980s, physical screening of the hip had many of the problems mentioned above, and routine x-rays could not address these problems. X-ray involves radiation exposure and cannot show most of a baby's hip joint, which is made of cartilage (22). Von Rosen viewed x-rays with each hip abducted 45° or more and rotated medially to effectively detect hip dislocation (7); however, this position is difficult to obtain, and positioning and reading should be the responsibility of trained specialists. When the position does not follow the standards, the results are easily biased (15). The clinical use of arthrography provides a diagnostic method that exposes patients to x-rays and is invasive. The cost of magnetic resonance imaging (MRI) is high, and the time needed to perform an investigation can be as long as 20 min, which requires sedation. In 1980, Reinhard Graf (23), an Austrian orthopedic surgeon, published an original paper on examinations of the infant hip using ultrasound. Graf showed that the anatomy of the infant hip joint, due to the predominantly cartilaginous femoral head, could be clearly visualized by ultrasound. In 1984, Graf (24) published an article analyzing the use of ultrasound in DDH screening and put forward the Graf diagnostic criteria, which became a milestone in the early diagnosis of DDH. Since then, ultrasound examination has gradually become one of the most important and frequently applied tools in the early diagnosis of DDH. In 2003, Roposch (25) commented that the introduction of hip ultrasound for DDH was a significant event, and similar examples are difficult to find in the field of pediatric orthopedics.

**Figure 1:**
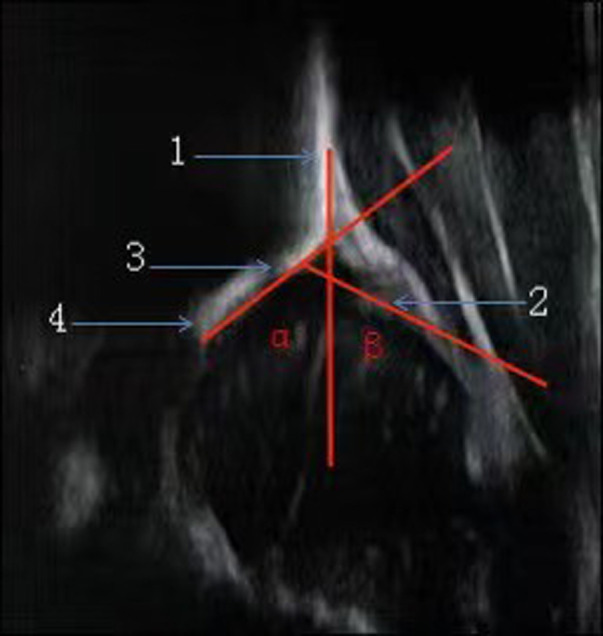
Accurate measurement of the hip joint is based on three intersecting lines, which define two angles (α & β). 1. Base line. 2. Acetabular Labrum. 3. Bony rim. 4. Lower limb of llium.

The Graf hip ultrasonography skill was the first method described and is perhaps the most widely used method. It is based on morphological evaluation of the resting hip in the coronal plane, and its primary aim is to assess acetabular morphology using a standardized approach. In addition to the standard approach, which is of paramount importance, clearly defined quality criteria, including anatomical identification (checklist I) and usability check (checklist II), are mandatory. Checklist I includes: (1) The chondro-osseous border; (2) The femoral head (the ossification nucleus; (3) The synovial fold; (4) The joint capsule; (5) The labrum; (6) The hyaline cartilage of the roof of the Acetabulum; (7) The bony part of the roof of the acetabulum; (8) The bony rim. Checklist II includes: (a) The presence of the lower limb of the os ilium, thus proving that the beam goes through the deepest part of the iliac bone in the acetabular fossa; (b) The straight silhouette of the iliac bone; (c) The labrum, thus proving that the scan has been performed in a standard plane (26, 27, 28). According to the Graf ultrasonographic hip classification system, the *α* and *β* angles are quantitative indicators of the bony and cartilage acetabular roofs (Figure 1), respectively, and infant hip joints are divided into 4 types and 10 subtypes (Table 1) (26).

**Table 1:** Sonographic hip types according to graf (26).

Type	Cartilage roof *α* angle	Cartilage roof *β* angle	Bony rim	Subtype
Type I Mature hip	Good *α* ≥ 60°	Covers the femoral head *β* < 77°	Angular/blunt	Ia: *β* ≤ 55° Ib: *β* > 55°
Type IIa Physiologically immature (age ≤ 3 months)	Deficient *α* = 50°–59°	Covers the femoral head *β* > 55°	Rounded	IIa+: *α* = 55°–59° (0–6 weeks) IIa−: *α* = 50°–54° (6–12 weeks)
Type IIb Delay of ossification (age > 3 months)	Deficient *α* = 50°–59°	Covers the femoral head β > 55°	Rounded
Type IIc Critical hip	Severely deficient *α* = 43°–49°	Still covers the femoral head β < 77°	Rounded to flattened
Type D Decentring hip	Severely deficient *α* = 43°–49°	Displaced β > 77°	Rounded to flattened
Type III Dislocated hip	Poor *α* < 43°	Pressed upwards, perichondrium slopes cranially	Flattened	IIIa: hypoechoic cartilage acetabular roof IIIb:hyperechoic cartilage acetabular roof
Type IV Dislocated hip	Poor *α* < 43°	Pressed downwards, perichondrium is horizontal or dips caudally	Flattened	

### Characteristics of the graf method compared with other ultrasonography methods for DDH

From 1980 to 2000, several scholars introduced new ultrasound methods for the assessment of infant hips. Differences among these methods were evident in the imaging planes used and the views obtained. The methods include Morin's method (29) and Terjesen's method (30), which focus on femoral head coverage, the Novick method (31) and Hacke method (2), which focus on detecting dynamic hip stability, Suzuki's method (32), which evaluates the relative position of the femoral head and acetabulum, and Rosendahl's method (33), which comprehensively evaluates morphology and stability. Harcke and coworkers (2) published a method combining static and dynamic hip ultrasound. The infant was imaged in the supine position with and without the use of stress (the Barlow maneuver), and the imaging focused mainly on the position of the femoral head at rest and during stress testing. The dynamic portion of the examination identifies whether the hip is stable, lax, subluxable/subluxed or dislocatable/dislocated and is easily affected by subjective factors related to the examiners. The Suzuki method (32) images both sides of the hip joint at the same time with a special long probe, judges the relationship between the femoral head and acetabulum by the auxiliary line. The author describes that the method is applicable to the infant in a harness or a plaster cast to demonstrate maintenance of reduction of a dislocated hip. However, based on the original illustration of the work, this is conceivable for a Pavlik harness, but seems difficult to imagine for a plaster cast that extends typically to the navel. Moreover, this technique does not consider the morphology of the acetabulum and the dynamic stability (34). The Terjesen method (30) is focused on the bony rim percentage (BRP), and the following distances are measured: (a) from the acetabular floor to the lateral bony rim of the acetabular roof and (b) from the same point on the acetabular fossa to the lateral joint capsule. The BRP is defined as a/b × 100. This method indirectly evaluates the development of the acetabulum according to the femoral head coverage but cannot classify hips in more detail and can only roughly reflect whether the hip is abnormal. Nevertheless, the Graf method is a direct evaluation of the development of the acetabular crest and can identify hip joints that need treatment through objective standards. Diaz et al. (35) and Peterlein et al. (36). assessed newborn hips with the Graf, Harcke, Suzuki, and Terjesen methods and found better reliability for the *α* angle than the *β* angle and the Graf technique to be the most reliable. According to some clinical studies, the main advantage of the Graf method is that it is a simple, accurate, quantitative and standardized examination associated with a clear standardized hip classification system that can directly guide treatment.

## Selective vs. Universal ultrasound screening

The DDH screening strategy has gradually changed since ultrasound was introduced for hip examinations in the 1980 s in many countries. Ultrasound screening can be “selective” for high-risk groups of neonates or “universal” for all neonates. Regarding the choice of selective or universal ultrasound screening, the evidence in the literature varies widely. To some extent, the literature reflects an evolution of understanding over time but also variation in practice among different countries or regions.

### Selective ultrasound screening program

Selective ultrasound screening is a combination of clinical and sonographic neonatal hip screening, which is performed on infants with hip abnormalities detected by physical examination and infants who have risk factors for DDH. Some studies have indicated that the main risk factors for DDH are breech presentation and a positive family history (8, 37, 38). Other risk factors include female sex, oligohydramnios, torticollis, and swaddling of the newborn (39–41).

Selective ultrasound screening is practiced in many countries, including North America, the United Kingdom (UK) and Australia (5, 39, 42). Most medical centers use a selective screening program based on risk factors and clinical examination; neonates with a positive examination undergo ultrasound scanning within two weeks of life, and infants at risk but exhibiting no clinical abnormalities undergo ultrasound within the first six weeks of life (8, 22, 43). If ultrasound is performed too early, some babies with transient immature and physiologically unstable hip joints may be diagnosed as positive cases. Therefore, it is important to determine the optimal timing of ultrasound examination/screening for DDH to prevent unnecessary repeat ultrasound examinations and treatments. The American Academy of Pediatrics (AAP) suggests that it is better not to perform hip ultrasound examinations within 2 weeks after birth. If necessary, they should be performed at 3–4 weeks, which is also recommended by the American Institute of Ultrasound in Medicine (AIUM). The American Academy of Orthopedic Surgeons (AAOS) recommends that an ultrasound be performed at 2–6 weeks after birth (43). Because most hip joints that initially appear immature will mature later, almost 90% of cases of mild hip instability at birth resolve spontaneously within the first eight weeks after birth (44). Gokharman and coworkers (45) also reported that an ultrasound scan performed at eight weeks after birth can safely and correctly predict the presence of any pathology and prevent unnecessary recurrent examinations and parental anxiety.

There is disagreement as to whether selective US screening strategies can reduce the incidence of late-detected DDH. Lewis and coworkers (46) reported a marked decrease in the number of late-diagnosed DDH patients, from 2.2 ‰ to 0.34 ‰ of newborns, using only selective ultrasound screening, which included 15% of the population with risk factors. This position was further supported by studies from other scholars (42, 47, 48). However, some reports declared that selective ultrasound cannot decrease the incidence of late-detected DDH, and most DDH is diagnosed in infants without any identifiable risk factors (8). There are some opinions that selective ultrasound screening depends on the experience of examiners, and even experienced specialists have been reported to misdiagnose DDH in 14% of patients diagnosed by ultrasound (49). A study by Sink et al. (50) showed that when children have no major risk factors for DDH and no positive physical examination results, they are not included in the group that is recommended to undergo selective ultrasound examination, which leads to false negatives. Their misdiagnosis rate can be as high as 85.3%. Delayed diagnosis of DDH likely leads to residual deformities, causes more damage, and results in higher surgical treatment costs. It is recommended that universal ultrasound screening be used when selective ultrasound screening does not reduce the incidence of late-detected DDH (50).

### Universal ultrasound screening program

Universal ultrasound screening involves performing hip ultrasound examinations on all newborns and is practiced in several countries, especially European countries such as Germany, Austria and Norway. However, the timing of the first ultrasound examination varies among countries, and most exams are performed within several days after birth or at 4 to 8 weeks of age (8, 51, 52, 53). Infants with positive and suspected cases detected by ultrasound examination are referred to a specialist for further diagnosis. Many studies have reported good results for universal ultrasound screening. A nationwide universal ultrasound screening program for DDH using the Graf technique was introduced in Austria in 1992, and early diagnosis of DDH significantly reduced both open surgeries and closed reduction interventions (51, 54). In Germany, a screening program for DDH that included universal static ultrasound imaging for all children was started in January 1996, and von Kries R et al. (55) showed that in the five years after the implementation of universal ultrasound screening, the rate of surgical treatment for DDH decreased by approximately 52%. Treiber M et al. (52) reported that universal ultrasound screening has reduced the number of late-detected cases, shortened the treatment time, and decreased the number of hip surgical procedures and recommended universal screening for neonates in countries with a higher incidence of DDH.

Some researchers have challenged universal screening programs, questioning their role in overdiagnosis and treatment, and the cost-effectiveness and effectiveness of the universal screening is also a concern. In a study from Norway analyzing 4,245 newborns that underwent both clinical and ultrasound screening for DDH within the first one to three days of life, Olsen and coworkers (53) reported that the addition of universal ultrasound in clinical screening for DDH doubled the treatment rate without influencing the already low numbers of late-diagnosed cases. Roovers and Rosendah also reported that according to their retrospective research, universal ultrasound screening does not eradicate late cases of DDH and leads to overtreatment (56, 57). Several other authors proposed programs for identifying the optimal timing for ultrasound screening to correctly distinguish patients with DDH that resolved spontaneously and those who needed treatment, which is of great importance in preventing unnecessary treatment. Bialik and coworkers (58) reported on the results of universal screening and showed that it is possible to differentiate between babies who require treatment and those who experience spontaneous resolution by postponing the onset of treatment for DDH and that 90.4% of babies with hip joints with DDH were spared unnecessary treatment. Based on the maturation curve of the hip joint, the ultrasound should be done within the time window until the beginning of the 6th week (22), and the division of Graf type II hips into IIa (younger than 3 months) and IIb (older than 3 months) can serve to avoid missing the favorable treatment interval and prevent unnecessary treatment. More other studies also recommend that ultrasound should be carried out 6 weeks after birth at the latest (8, 51, 52, 59).

Another concern is whether the benefits of universal ultrasound screening justify the costs. Thaler et al. (60) performed a retrospective analysis comparing the number and cost of interventions due to hip dysplasia in three patient age groups and showed not only higher initial costs caused by ultrasound screening but also a significant reduction in the total number and overall costs of newborns with dysplastic hips undergoing operative and nonoperative treatment. Similar findings were reported by Clegg et al. (61), who compared the total cost of running the screening program and the expense of treating the condition using three screening methods: universal clinical examination alone, universal clinical examination with selective ultrasound examination for newborn babies with risk factors, and universal ultrasound screening alone; they concluded that the overall cost for DDH management is comparable for the different screening policies. The International Interdisciplinary Consensus Ultrasound Meeting on the Evaluation of DDH (8) held in 2018 strongly agreed that when all short- and long-term costs are taken into account, a system of universal ultrasound screening using the Graf technique is cost-effective and will result in a reduction in later problems related to dysplasia.

## Treatment

Although the optimal method to screen for DDH is controversial, the goals of different screening programs are the same, which are to prevent undiagnosed cases and allow for earlier, less aggressive interventions to achieve hip reduction. Treatment algorithms for hip dislocation vary widely internationally. Ultimately, all dislocated hips should be reduced as early as possible by diverse reduction bandaging, extension treatment, or manual closed reduction which can be facilitated by arthrographic control (62). A recent meta-analysis of 29 observational studies showed that dynamic splinting, including Pavlik harness, Frejka splint, or Tubingen splint, has a low contraindication for hip retention and is very well tolerated, and the Pavlik harness is still the most commonly used brace for dynamic splinting, but Tubingen splints have been shown to have better results with greater tolerance and compliance. Patients more than 6 months old with acetabular dysplasia but stable hip joints can be treated with a static brace, such as a rhino-style brace, an Ilfeld brace or a generic abduction brace, but the femoral head should be well centered in the acetabular base (63). It should be noticed that conservative banding treatment also carries the risk of femoral head necrosis, so a change in procedure is recommended after 2 to 3 weeks if reduction is inadequate (64). Hips that cannot be reduced closed can be reduced openly from about 6 months of age with or without femur-side and/or acetabular-side bony correction (65).

Well-planned systematic DDH screening guarantees screening quality and efficiency. The value of hip screening is dependent on adherence to the correct technique and a standardized system of teaching and training correct hip physical examination and ultrasound skills; therefore, a certification for operators dedicated to performing DDH screening would also be useful. Another concern is the need for health services to promote multidisciplinary collaboration so a consensus can be reached on various issues related to DDH screening and suitable centers for screening can be identified. The formulation of plans for early detection, diagnosis and treatment and an optimized screening system with a strict screening process and quality management should also be considered.

## Conclusions

There are great discrepancies in newborn hip screening programs among different countries and regions. Most of them have adopted universal or selective ultrasound screening strategies, and both types of strategies are used in combination with physical examinations. Hip ultrasonography in the method according to Graf is currently the most accurate diagnostic tool for DDH in early infancy and requires thorough screening plans and standardized hip ultrasound teaching and training programs. Abiding by a strict screening process and quality management are equally important. Universal ultrasound screening has a greater effect than other screening programs on reducing late-detected cases but may have a tendency toward overtreatment and high initial costs. By optimally timing the ultrasound exam and properly delaying treatment, overtreatment could be reduced. In the long run, a universal ultrasound screening strategy is more cost-effective, as the cost is offset by the costs of avoided surgery and nonsurgical treatment. In addition, it may reduce the occurrence of residual malformations of DDH. All these may be subjects of future investigations.
